# Remodeling of Embryo Architecture in Response to Vanadium and Increased Temperatures: From Morphometric to Molecular Changes

**DOI:** 10.3390/jox15010022

**Published:** 2025-02-01

**Authors:** Roberto Chiarelli, Chiara Martino, Rosaria Scudiero, Alessio Terenzi, Fabiana Geraci

**Affiliations:** 1 Department of Biological, Chemical and Pharmaceutical Sciences and Technologies (STEBICEF), University of Palermo, Viale delle Scienze, 90128 Palermo, Italy; chiara.martino@unipa.it (C.M.); alessio.terenzi@unipa.it (A.T.); fabiana.geraci@unipa.it (F.G.); 2 NBFC, National Biodiversity Future Center, Piazza Marina 61, 90133 Palermo, Italy; 3 Department of Biology, University Federico II, 80126 Napoli, Italy; rosaria.scudiero@unina.it

**Keywords:** embryo cytotoxicity, skeleton, gelatinases, metalloproteinases, global warming, climate change, metal pollution, marine ecotoxicology, marine heatwaves, biomineralization

## Abstract

The study of ecotoxicity induced by vanadium (V) represents an area of increasing interest due to the growing use of V in both the industrial and pharmaceutical areas. This leads to its introduction into water environments, marking a developing problem, especially since rising global temperatures appear to intensify its toxic properties. Cytotoxicological approaches carried out on whole marine embryos represent a valid research tool since they grow directly in contact with the pollutants and are equipped with highly responsive cells to stressors. Here, we discuss the detrimental impact on *Paracentrotus lividus* sea urchin embryos resulting from the combination of V and higher temperatures, reflecting the effects of climate variation. The results demonstrate the remodeling of embryonic architecture at the morphometric level, revealing developmental delays and anomalies. These malformations involve variations in the total skeletal mass due to the almost total absence of the skeleton, with the exception of small calcareous aggregates. Furthermore, both a modulation in total tissue remodeling enzymatic activities and a variation in the amount of three MMP-like gelatinases (MMP-2, -9, and -14) were observed. This research demonstrates that climate change significantly increases the harmful effects of V, emphasizing the necessity for comprehensive toxicity assessments in environmental evaluations.

## 1. Introduction

In recent years, research on vanadium (V) compounds has intensified because of their industrial and therapeutic potential [1]. It has become evident that the increased use of this metal intensifies its environmental dispersion and bioaccumulation along the food chain [2], raising considerable concern as the understanding of its harmful effects on aquatic life remains limited [3].

Historically, the sea urchin embryo of the species *Paracentrotus lividus* has been used to study the toxic effects of metals on cell stress, survival, and death [4,5]. The choice of an embryonic experimental model over a single cell line gives the opportunity to study multiple effects at the level of a whole organism equipped with highly responsive cellular stress response mechanisms, thus representing a valid bioindicator of chemical pollution [6,7,8,9,10]. When exposed to polluting metals, the sea urchin embryo undergoes changes in its developmental phenotype [4,11,12], primarily affecting skeletal development in a detrimental way [13,14,15]. Since the skeleton is built mainly of calcium carbonate [13,16], polluting metals probably disturb the calcium uptake, which could explain why the skeleton undergoes drastic changes [14,17]. Experiments on the determination of the content of polluting metals in sea urchin embryos, such as Cd, Mn, Gd, and V, showed how their concentration increase is correlated with a reduction in intracellular calcium [12,13,14,18] and in total skeletal mass [13,19,20].

Sea urchin embryos respond to toxic metal exposure by activating a coordinated set of defense strategies, starting with cytoprotective responses such as the generation of heat shock proteins, and advancing to additional approaches like autophagy to clear out damaged proteins and organelles [19,20,21,22]. Distinct chemicals can activate particular apoptotic traits in sea urchin embryos, which are classified into two types: cell-selective apoptosis, where DNA fragmentation is limited to a select group of severely affected cells, acting as a protective mechanism for the embryo; and total apoptosis, where DNA fragmentation is pervasive across all cells, culminating in the embryo’s death [19].

Starting in 2021, the embryos of sea urchins have been used in experiments to define the toxicological properties of V [19]. *P. lividus* embryos were exposed to a wide range of sublethal V concentrations, ranging from environmental to cytotoxic (50 nM–1 mM), finding a strong dose-dependent response causing severe morphological malformations and the activation of the cellular stress response, incorporating both heat shock proteins and the autophagy mechanism [19]. Results also showed V bioaccumulation in a dose- and time-dependent manner and the inhibition of fertilization [17]. Since the morphological malformations caused by V mainly affected the skeleton and embryonic architecture, which is governed by proteolytic enzymes that degrade the extracellular matrix [17,18], a further study analyzed the proteolytic enzymes essential to promote cell movement, especially during gastrulation [17,18], finding that that V is able to modulate the expression of these enzymes acting in a dose/time-dependent manner [18].

The study revealed that different cytotoxic and environmental levels of V led to the modulation of nine proteases at the 36 h mark of the early pluteus stage [17]. Enzymatic activities related to proteolysis involve metal ion-dependent enzymes, notably metalloproteinases (MMPs), a category of proteinases that require zinc (Zn) and calcium (Ca) for their activity and are influenced by vanadium (V) in diverse organisms [18].

We provided insights into the MMP family, highlighting the specific data related to MMP-2, -9, and -14 in sea urchin embryos. For these MMPs, a modulation induced by V was reported [18]. Subsequent studies focused on two cytotoxic doses, 500 μM and 1 mM, to fully characterize the molecular defensome of *P. lividus* in response to V, showing ERK modulation and the activation of cell-selective apoptosis acting as an embryo survival strategy [21].

Even if the above-mentioned studies on V response clarify some of its mechanisms of action, they do not consider the concomitant presence of physical parameters related to environmental changes. Evidence suggests that climate change will modify the impact of pollution on aquatic life, resulting in different responses from bioindicator species. Specifically, fluctuations in seawater temperature can impact the harmfulness of metals, altering their accessibility and their role as bioactive substances [22,23,24,25,26,27,28,29].

Sea urchin embryos represent a suitable biomarker for testing temperatures across near-future projections (+3 °C, 21 °C) and present-day marine heatwave conditions (MHWs: +6 °C, 24 °C) [20]. Up to the present, the number of studies that have analyzed the effects of both chemical pollutants and climate change on *P. lividus* embryos is quite small. It was found that at a physiological temperature of 18 °C, gadolinium (Gd) pollution negatively impacted the growth of sea urchin embryos and their skeletons. Interestingly, raising the temperature to 21 °C alleviated some of these harmful effects, but increasing it to 24 °C resulted in compounded negative consequences [20]. Similarly, a higher sensitivity to other chemical pollutants, such as chlorpyrifos and microplastics, under ocean warming and acidification conditions was reported [30].

In a recent study, we examined how exposure to 1 mM vanadium (V) and conditions associated with global warming affect the development of *P. lividus* sea urchin embryos. Our findings revealed an increase in malformations, hindered skeletal development, activation of cellular stress responses and apoptosis, as well as a time- and temperature-dependent rise in V bioaccumulation, alongside a decrease in calcium levels [31]. Here, we expand our previous results by studying how 1 mM V exposure together with warming at near-future projections (21 °C) or at actual marine heatwave conditions (24 °C) could alter embryo architecture at the morphometric and the molecular level, investigating variations in the total skeletal mass and in the activities of total gelatinases and three MMP-like gelatinases (MMPs-2, -9, and -14).

Examining the relationship between metal pollution and climate change can enrich our insights into the adaptative responses of bioindicator species, which may inform environmental management decisions for policymakers.

## 2. Materials and Methods

### 2.1. Embryos Cultures, Treatments, and Morphological/Morphometric Analyses

Adult specimens of *P. lividus* sea urchins were harvested from the west coast of Sicily, in the Favignana island. Adult acclimation was carried out maintaining male and female specimens in aquaria at 18 °C (±0.3 °C) until the gamete collection phase.

At least 12 female individuals and 12 male individuals were selected for each experimental replicate. Eggs were fertilized with an aliquot of sperm from a mixture obtained from each male individual in order to obtain maximum variability.

For both types of gametes, quality was tested according to criteria related to microscopy observation. Specifically, only eggs with a perfectly spherical morphology and those for which a rapid fertilization test [17] gave a positive result, approximately 99% of fertilization, were selected. The quality of the spermatozoa was tested by observing morphology and motility. Then, 5000 eggs/mL were fertilized using 10 µL of semen, diluted in 1 mL MFSW.

Three separate samples of embryo cultures were cultivated at varying temperature conditions: 18 °C, 21 °C, and 24 °C, reflecting physiological, near-future projections, and present-day heatwave conditions, respectively. At the same time, three samples of embryo cultures maintained at identical temperatures underwent exposure to 1 mM sodium orthovanadate (Na_3_VO_4_, from now on V) (Sigma-Aldrich, S6508, Waltham, MA, USA).

Stock solution (0.1 M) was prepared to ensure the presence of vanadate monomers, avoiding the presence of decavanadate, as reported by Chiarelli et al. [17].

Three replicates per condition were tested. Embryo development was performed using three thermostatic chambers. All embryonic cultures were carried out in sterilized Pyrex glass containers (250 cm^3^ volume) containing 150 mL of culture in Millipore-filtered seawater (MFSW) buffered by 1M HCl Tris at pH 8.0.

After 24 and 48 h, microscopy analyses were performed using an Optika microscope (Optika, Ponteranica, Italy), under a 10× objective equipped with a digital camera (Nikon Sight DS-U1, Tokyo, Japan). The selected concentration of vanadium aligns with the levels previously employed to investigate the toxicological pathways and the molecular reactions triggered, representing the minimum concentration that results in complete developmental anomalies [31].

A total of around 100 embryos per condition underwent analysis and were classified according to predefined standards [31].

Morphometric analyses were carried out according to criteria described previously [17,18].

### 2.2. Skeleton Analysis

The skeleton of the larvae was isolated after 48 h of development and treated as reported by Chiarelli et al. [19].

For each treatment, about 100 whole skeletons were photographed 48 h post-fertilization and, for each skeletal spicule, BR (body rod), PO (post-oral arm), and Pre-O (pre-oral arm) length was measured using the ImageJ Fiji analyzing software (https://fiji.sc/, (accessed on 1 October 2024)).

### 2.3. Zymography

Pellets from embryos and larvae at 24 and 48 h of development were obtained from the cultures by centrifugation at 1500 rpm (VWR galaxy 7D, Milan, Italy) and stored at −75 °C until use. Lysis was performed by using a buffer (20 mM Tris, pH 7.4; 150 mM NaCl; 0.5% Triton X-100) without protease inhibitors in order to avoid blocking enzymatic activities. Samples were lysed by three cycles of freezing (liquid nitrogen) and thawing (37 °C).

After centrifugation at 14,000 rpm (Eppendorf centrifuge 5430 R, Hamburg, Germany) for 30 min at 4 °C, supernatants were stored at −75 °C until use. For zymography, 15 µg of proteins for each sample was separated by 10% SDS-PAGE gel as described previously [17].

Proteolytic enzymatic activities, corresponding to gelatinolytic bands, were quantified using the ImageJ Fiji analyzing software.

### 2.4. Electrophoretic Analysis and Immunoblotting

Pellets were recovered from 5 mL of embryo cultures at 24 and 48 h of development and were homogenized using a lysis buffer as described by Chiarelli et al. [18,19], and 30 μg of sample was separated by 10% SDS-PAGE.

Bands were detected using the ECL Plus Western Blotting Detection system™ (Amersham) through the molecular imager VersaDoc MP Systems (Bio-Rad, Hercules, CA, USA).

Band intensity analysis was carried out using the Quantity One software (version 4.6.6, Bio-Rad), which designated actin bands as the reference for loading control.

### 2.5. Statistical Analysis

This research utilized the statistical analysis techniques detailed by Martino et al. [20]. All data were analyzed using Statistica 13.2 software (StatSoft, Tulsa, OK, USA), with *p* ≤ 0.05 as the level of significance. Two-way analysis of variance (ANOVA) was carried out with V concentration and temperature as fixed factors. Tukey’s HSD test was used as a post-hoc test for mean comparison, and homogeneity of variance was checked and confirmed using Levene’s test.

## 3. Results

### 3.1. V and Increased Temperatures Induce Morphological and Volumetric Alteration of the Embryos

**Figure 1:**
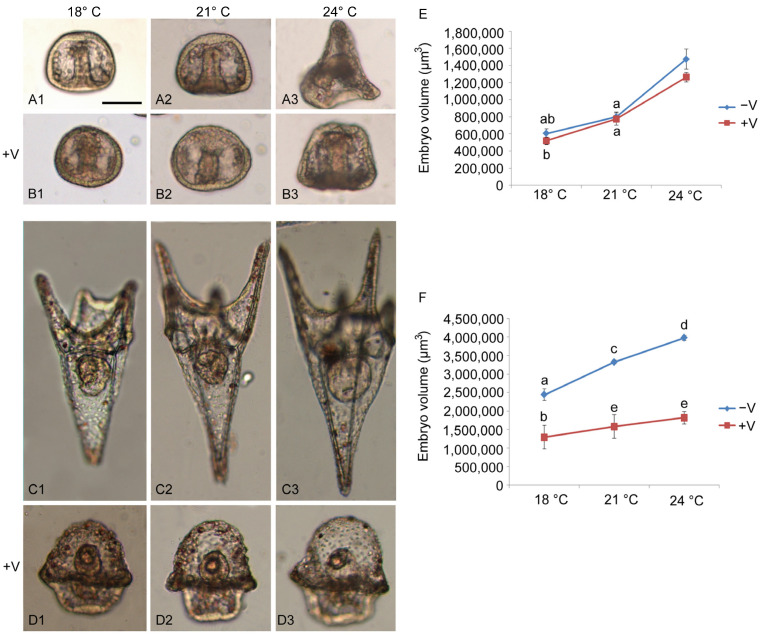
Morphological and morphometric analysis of *P. lividus* embryos. Images representative of embryos and larvae at 24 and 48 h of development. Embryos were grown at three different temperatures: 18 °C (**A1**,**B1** and **C1**,**D1**), 21 °C (**A2**,**B2** and **C2**,**D2**), and 24 °C (**A3**,**B3** and **C3**,**D3**) with (**B1**–**B3** and **D1**–**D3**) or without V (**A1**–**A3** and **C1**–**C3**). Bar: 100 μm. The line graphs (**E**,**F**) show data related to the volumetric analysis. Each set of experiments was replicated three times, and the data are reported as mean values with standard deviation (*n* = 3). Treatments with the same letter do not differ in Tukey HSD results.

We exposed embryos to V, increased temperature from fertilization onwards, and examined the morphometric effects at two key stages of embryogenesis: gastrula (24 h), which marks the beginning of skeletal formation and tissue differentiation, and pluteus (48 h), the larval stage, which highlights the presence of specialized tissues and organs (Figure 1A–D).

After 24 h of development/treatment, there was a drastic effect induced by V coupled with increased temperatures. At physiological temperature (18 °C), embryos exposed to V had a slight delay in development, being at the early gastrula stage while the controls were advanced gastrulae (Figure 1(A1,B1)).

The volumetric analysis carried out after 24 h of development showed, for both unexposed and V-exposed embryos, an increase in their volume as a function of temperature rise (F_2,15_ = 205.6, *p* < 0.005) and V exposure (F_1,12_ = 10.11, *p* < 0.05). The volume of V-untreated embryos was only slightly higher than that of V-exposed embryos for all the tested temperatures (Figure 1E). No interaction between these two factors was found.

A similar result was obtained after 48 h of development/treatment, with a temperature-dependent increase in embryo volume (F_2,15_ = 3.6, *p* = 0.05). On the contrary, the volume of V-unexposed embryos was always higher than that of exposed embryos (F_1,16_ = 14.9, *p* < 0.05) for all the examined temperatures (Figure 1F). No interaction between these two factors was found.

### 3.2. V and Increased Temperatures Induce Alterations of Total Skeletal Mass of the Embryos

**Figure 2:**
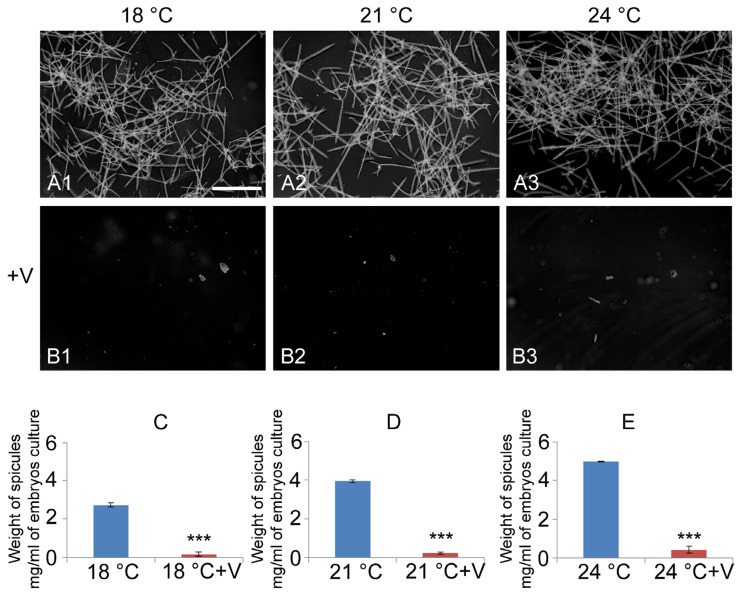
Skeleton analysis. Images show representative fields, captured by light microscope, of purified spicules after 48 h of development from untreated (**A1**–**A3**) and V-treated embryos (**B1**–**B3**). Embryos were grown at three different temperatures: 18 °C (**A1**–**B1**), 21 °C (**A2**–**B2**), and 24 °C (**A3**–**B3**) in the presence (**B1**–**B3**) or absence (**A1**–**A3**) of V. Bar: 100 μm. Line graphs (**C**–**E**) show data related to average weight spicules, expressed in mg per mL of embryo culture. Each set of experiments was replicated three times, and the data are reported as mean values with standard deviation (*n* = 3). Statistical analysis was performed using *t*-tests with *** *p* ≤ 0.0005.

To investigate how V, in combination with temperature rise, affects embryo biomineralization, we treated them since fertilization and examined their combined effects after skeletogenesis at 48 h of development (Figure 2).

At all developmental temperatures (18 °C, 21 °C, and 24 °C) V caused the blockage of biomineralization (F_1,16_ = 525.5, *p* < 0.005). Only a few calcareous aggregates have been observed in V-treated embryos. This aspect highlights that the primary mesenchyme cells (skeletogenic cells) survived the treatment and are still functional but failed to activate the correct synthesis of the skeletogenic matrix since it was blocked by V presence (Figure 2(B1–B3)).

Interestingly, there was a significant temperature-dependent effect on the total skeletal mass (F_2,15_ = 220.2, *p* < 0.005). Microscopy observations showed that increasing temperature resulted in longer and thicker spicules (Figure 2(A1–A3)). Quantitative analysis of total skeletal mass (histograms in Figure 2) showed how temperature is a positive factor for the biomineralization process. We also found a significant effect of the interaction between V and temperature (F_5,12_ = 143.5, *p* < 0.005).

**Figure 3:**
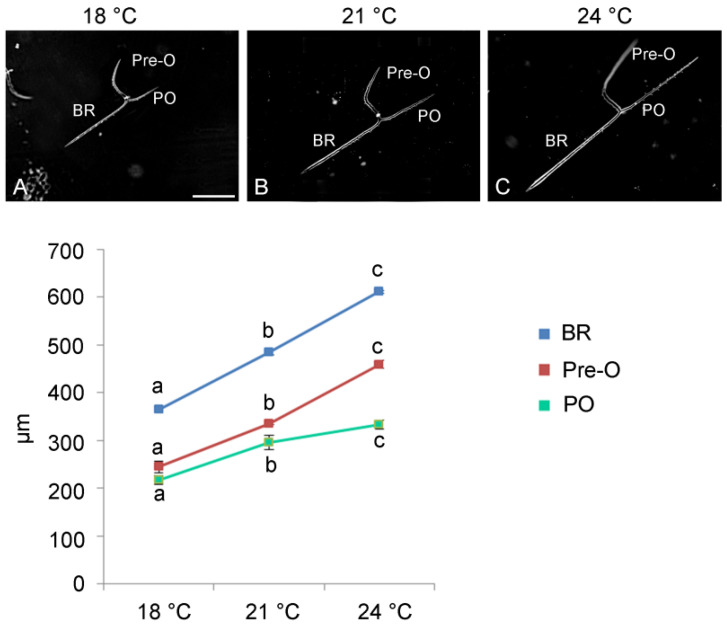
Morphometric evaluation of the length of BR (body rod), PO (post-oral arm), and Pre-O (pre-oral arm). Images of representative spicules purified from control embryos at 48 h of development, observed under light microscopy (20X), from V-untreated embryos (**A**–**C**) developed at three different temperatures: 18 °C (**A**), 21 °C (**B**), and 24 °C (**C**). Bar: 100 μm. The line graphs show data related to the average of measurements. Each set of experiments was replicated three times, and the data are reported as mean values with standard deviation (*n* = 3). Treatments with the same letter do not differ in Tukey HSD results.

This study involved a quantitative evaluation of larvae cultivated without V at three varying temperatures, analyzing three key morphometric parameters related to skeletogenesis. This investigation was conducted on the individual skeletal element, represented by the triradiate spicules of calcium carbonate, and showed the significant temperature-dependent increase of all the three microanatomical traits considered (PO: F_2,6_ = 83.2, *p* < 0.005; BR: F_2,6_ = 4981.5, *p* < 0.005; and Pre-O: F_2,6_ = 457.3, *p* < 0.005) (histograms in Figure 3). This skeletal element displays the following microanatomical traits, which are correlated with the trophic capacity of the organism and whose variation in length can give an indication of the skeletal capacity of the embryo: the BR (body rod); the PO (post-oral arm), and the Pre-O (pre-oral arm) (Figure 3A–C).

### 3.3. Total Proteolytic Activity Reflects the Status of Remodeling of the Extracellular Matrix

As outlined by Chiarelli et al., V can impact the embryonic development of sea urchins by hindering their maturation and altering the structure of their skeletal systems. [19]. Furthermore, it has also been demonstrated that V influences MMP activity, depending on metal concentration and developmental stages [17,18]. The expression of these enzymatic activities is also involved in sea urchin embryo skeletogenesis [17].

To study how V, in combination with temperature increase, affects the remodeling of the extracellular matrix, a zymographic analysis was performed to detect gelatinase activities at 24 and 48 h of development, finding a variable pattern of proteolytic activities.

As previously demonstrated by Chiarelli and collaborators in 2022 [17], in sea urchin embryos there are nine different gelatinases (named according to their apparent molecular masses as 309, 255, 177, 79, 59, 34, 30, 25, and 22).

**Figure 4:**
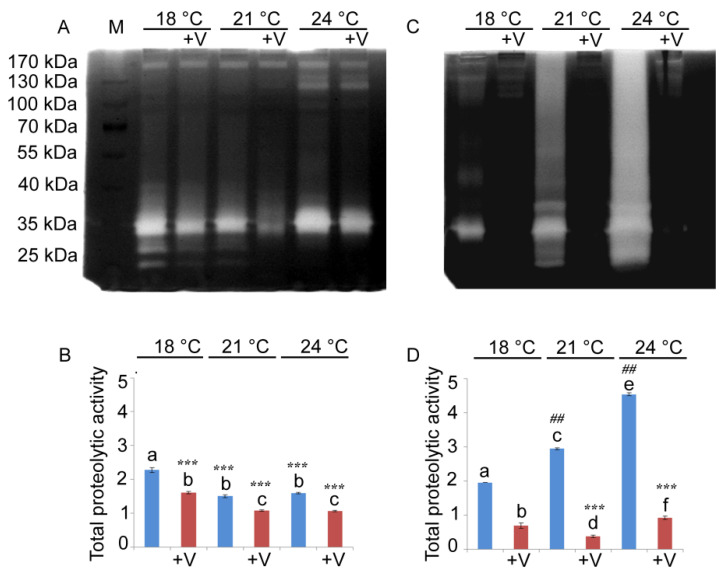
Metal-related enzymatic activities analyzed by gelatin zymography. (**A**) Zymograms showing bands in lysates of untreated and V-treated embryos grown at 18 °C, 21 °C, and 24 °C for 24 h and (**C**) 48 h. (**B**) Line graph reporting the modulation over time of the total proteolytic activity for 24 h and (**D**) 48 h. Each set of experiments was replicated three times, and the data are reported as mean values with standard deviation (*n* = 3). (**B**) *** *p* < 0.0001 compared with untreated embryos grown at 18 °C. (**D**) *** *p* < 0.0001 compared with untreated embryos grown at 18 °C and ## *p* < 0.005 compared with V-treated embryos grown at 18 °C. The band intensity of panels (**A**,**C**) was measured by ImageJ 1.46r image analyzing software. Treatments with the same letter do not differ in Tukey HSD results.

After 24 h of development, V (F_1, 16_ = 850.6, *p* < 0.0001), temperature (F_2, 15_ = 512.5, *p* < 0.0001), and their interaction (V × temp: F_5, 12_ = 14.5, *p* < 0.005) influenced the total proteolytic activity. V-treated embryos showed a reduction in total proteolytic activity compared to embryos not exposed to V at all three developmental temperatures. This reduction was approximately 1.4-fold for 18 °C and 21 °C and 1.5-fold for 24 °C (Figure 4B). Furthermore, it was observed that compared to embryos grown at physiological temperature (18 °C), both V-treated and untreated embryos grown at higher temperatures (21 °C and 24 °C) showed a significant reduction in total proteolytic activity (Figure 4B).

It is also important to note that, in addition to the modulation of total proteolytic activity, the different developmental temperatures affect the increase/decrease of specific gelatinase activities, both in V-treated and untreated embryos.

Embryos grown at 18 °C showed gelatinolytic bands of 177, 34, 30, 25, and 22 kDa. The major decrease in proteolytic activity in V-exposed embryos occurs in all the low molecular weight enzyme activities (i.e., 34; 30; 25, and 22 kDa) (Figure 4A). The reduction regarding these four molecular weights gelatinolytic activities was about 3.1-fold. There was no appreciable reduction in the band of 177 kDa (Figure 4A).

Gelatinolytic enzymes with the same molecular weights as those identified in embryos developed at 18 °C were also observed when the growing temperature was 21 °C. Similarly to 18 °C, the major diminution in proteolytic activity in embryos exposed to V was observed for 34 and 30 kDa enzyme activities (about 3.8-fold). On the contrary, the lowest molecular weight activities (25 and 22 kDa) were completely inhibited (Figure 4A).

A different result was obtained when embryos were grown at 24 °C. Indeed, they showed proteolytic activity related to the 177 kDa enzyme, but in addition, two new proteolytic activities (125 and 122 kDa respectively) were observed. Furthermore, the two low molecular weight proteolytic activities (25 and 22 kDa) totally disappeared. In embryos exposed to V, compared to untreated embryos, no specific reduction was observed in one or more particular proteolytic activities, but the decrease was proportionally distributed to all enzymatic activities (Figure 4A).

At 48 h of development, V-treated embryos showed a drastic reduction in the total proteolytic activity in comparison with untreated embryos (F_1, 16_ = 14,285.5, *p* < 0.0001), at all the developmental temperatures tested (Figure 4D). This reduction was about 2.8-fold for 18 °C, 7.9-fold for 21 °C, and 4.9-fold for 24 °C. Conversely, untreated embryos exhibited an increase in total proteolytic activity as a function of temperature rise (F_2, 6_ = 1664.2, *p* < 0.0001) (Figure 4D). There was also an effect caused by the interaction of the two factors (V × temp: F_5, 12_ = 1075.2, *p* < 0.0001).

As observed after 24 h, at this developmental stage, in addition to the modulation of total proteolytic activity, temperature also affected the increase/decrease of specific gelatinase activity, both for V-treated and untreated embryos.

In embryos grown at 18 °C, gelatinolytic activity of the following molecular weights was observed: 177, 125, 34, and 30 kDa. The major reduction in total proteolytic activity in embryos exposed to V occurs because all low molecular weight enzyme activities (34 and 30 kDa) were no longer expressed.

Embryos grown at 21 °C showed all the proteolytic enzymes, with a prevalent expression of the 34, 30, 25, and 22 kDa proteins. In addition, another band was detected at 38 kDa (Figure 4C). These low molecular weight proteolytic activities disappeared in V-treated embryos, justifying the reduced gelatinolytic activity. A very faint signal was observed at 177 and 125 kDa (Figure 4C).

Embryos grown at 24 °C showed the highest level of proteolytic activity, with a major signal for 38, 34, 30, 25, and 22 kDa enzymes (Figure 4C). In embryos exposed to V, compared to those unexposed, the type of expressed enzymes was the same as the V embryos at 21 °C (Figure 4C).

All these data demonstrated that during development, V-treated and untreated sea urchin embryos exposed to various temperatures exhibited a different pattern of expression and a different level of proteolytic activities. In particular, a more detailed analysis of the proteolytic bands indicated that there was a modulation of specific extracellular matrix degradation enzymes.

### 3.4. Metalloproteinases-2, -9, and -14-like Protein Modulation

**Figure 5:**
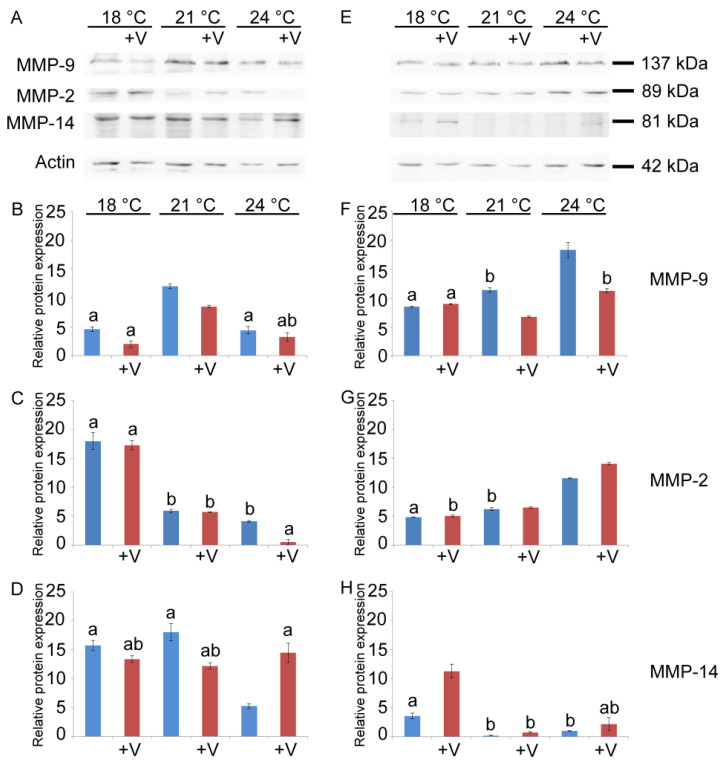
Immunoblotting and quantitative analysis for MMPs. (**A**,**E**) Total lysates of untreated and V-treated embryos grown at 18 °C, 21 °C, and 24 °C after 24 h (**A**) and 48 h (**E**) of development, immunoreacted with anti-MMP-9, -MMP-2, and -MMP-14 antibodies. Histograms (**B**–**D**,**F**–**H**) showing the densitometric analysis of the obtained bands. Relative protein expression, reported as arbitrary units, was calculated as MMP band density normalized to the corresponding intensity of actin. Each set of experiments was replicated three times, and the data are reported as mean values with standard deviation (*n* = 3). Treatments with the same letter do not differ in Tukey HSD results.

We have previously performed a partial biochemical characterization of gelatinases, showing that some of them are members of the MMP family [17]. Given the structural similarities with matrix metalloproteinases and the conservation observed among family members, we performed immunoblotting with antibodies targeting MMP-2, MMP-9, and MMP-14. This investigation sought to evaluate the influence of temperature and V on the activity of these specific metalloproteinases (Figure 5A,E).

At 24 h, V (F_1, 16_ = 96.5, *p* < 0.0001), temperature (F_2, 15_ = 335.7, *p* < 0.0001), and their interaction (V × temp: F_5, 12_ = 7.9, *p* < 0.05) had a significant effect on MMP-9 levels. Unexposed embryos showed an increase in the MMP-9-like protein level of about three times if the temperature was increased from 18 °C to 21 °C. However, when the temperature increased to 24 °C, the levels of MMP-9 remained comparable to those of the physiological development temperature (18 °C) (Figure 5B). On the contrary, at 18 °C and 21 °C, the level of the MMP-9-like protein in V-treated embryos was lower (about 2.2- and 1-4-fold, respectively) if compared to embryos grown at the same temperature without V (Figure 5B). There was a significant 4.2-fold increase in the MMP-9-like protein levels between 21 °C and 18 °C in V-exposed embryos (Figure 5B). At 48 h, the MMP-9-like protein was also modulated by V-exposure (F_1, 16_ = 177.3, *p* < 0.0001), increased temperature (F_2, 15_ = 195.9, *p* < 0.0001), and their interaction (V × temp: F_5, 12_ = 62, *p* < 0.0001) (Figure 5E,F). In V-treated embryos, at 18 °C, the MMP-9-like protein level was similar to the control, while at 21 and 24 °C, the level of this protein was lower (about 1.7-fold) than in untreated embryos grown at the same temperatures (Figure 5F). Comparing V-exposed embryos, MMP-9-like protein level decreased at 21 °C (about 1.7-fold) and increased at 24 °C (about 1.2-fold) compared to the corresponding embryos exposed to V but grown at physiological temperature (Figure 5F).

At 24 h, an MMP-2-like protein showed to be modulated by temperature variations (F_2, 15_ = 745.5, *p* < 0.0001), V (F_1, 16_ = 19.4, *p* < 0.0001), and their interaction (V × temp: F_5, 12_ = 9.5, *p* < 0.005) (Figure 5C). A drastic reduction, inversely proportional to temperature increase, occurred at 21 °C and 24 °C in embryos grown either with or without V (Figure 5C). At 18 °C and 21 °C, no difference was observed in the MMP-2-like protein levels between V-treated embryos and controls. On the contrary, at 24 °C, its amount in V-treated embryos was drastically reduced (about 7-fold) compared to untreated embryos grown at the same temperature (Figure 5C). After 48 h, MMP-2 was also modulated by exposure to V (F_2, 16_ = 211.5, *p* < 0.0001) and increased temperature (F_2, 15_ = 4703.1, *p* < 0.0001), as well as by their interaction (V × temp: F_5, 12_ = 116.5, *p* < 0.0001) (Figure 5E,G). At 18 °C and 21 °C, there was no significant variation between V-treated and untreated embryos. Conversely, at 24 °C, the MMP-2-like protein increased in V-treated (about 1.2-fold) compared to untreated embryos (Figure 5G).

At 24 h, MMP-14-like protein showed to be modulated by temperature (F_2, 15_ = 46.5, *p* < 0.0001) and its interaction with V (V × temp: F_5, 12_ = 86.8, *p* < 0.0001), but not by V exposure. At 21 °C, the level of MMP-14-like protein in V-treated embryos was 1.5-fold lower than in untreated embryos, while at 24 °C it was 2.8-fold higher in V-treated embryos than in untreated embryos grown at the same temperature (Figure 5D). Unexposed embryos had comparable levels of MMP-14-like protein at the lowest temperatures (at 18 °C and 21 °C), while at 24 °C its amount was drastically reduced (Figure 5D). On the other hand, in V-exposed embryos, the level of this MMP was comparable at all treatment temperatures. At 48 h, the MMP-14-like protein was modulated by temperature (F_2, 15_ = 171.6, *p* < 0.0001), V (F_1, 16_ = 91.8, *p* < 0.0001), and their interaction (V × temp: F_5, 12_ = 48.4, *p* < 0.0001). Without V, an appreciable level of MMP-14-like protein was observed only at 18 °C, while in all the other cases, this protein was almost absent (Figure 5E,H). In V-exposed embryos grown at 18 °C, there was a significant 2.3-fold increase when compared to untreated embryos (Figure 5H).

## 4. Discussion

V represents an emerging pollutant due to its increased use in industrial processes and as metallodrug. At the same time, it has been widely demonstrated that the ocean is undergoing progressive warming, with MHWs reaching peak temperatures by up to +6 °C or more [32].

This study presents evidence demonstrating the detrimental effects of the interaction between vanadium and elevated temperatures on the morphological characteristics of sea urchin embryos. Moreover, since it has been already proved that V induces a variation in the internal concentration of several metals, especially Ca [31], we extended our research to the study of enzymatic activities that use metals as a cofactor, both through zymography assays and analysis of metalloproteinase levels.

### 4.1. Increased Temperatures Amplified the V-Cytotoxicity in Terms of Biometry in Sea Urchin Embryos

Research has shown that V causes morphological changes in sea urchin embryos and rising temperatures exacerbate its toxic effects, leading to a higher incidence of malformations in the embryos [31].

It is well known that embryo growth rate is a function of temperature [33] and in this study, this dependence is supported by the increase in their volume. On the contrary, the presence of V as a second stressor, in addition to inducing morphological anomalies and developmental delays, provoked a reduction in the volume of the embryos.

Therefore, our results suggest an alteration in embryonic growth that could derive from a change in their metabolism. In fact, it has been already reported that in *Crossostrea virginia*, the presence of combined stress (i.e., Cd and elevated temperatures) affects mitochondrial metabolism [22]. The morphological alterations detected in sea urchin treated embryos do not seem to be randomly distributed in embryo districts, but are rather tissue-specific, as highlighted in other species of marine organisms subjected to the double metal/temperature stress [29].

### 4.2. Biomineralization Is Affected by V and Increased Temperatures

Development and biomineralization are important markers to determine the impacts of environmental stressors on sea urchin species. Marine invertebrates experience notable developmental benefits from temperature increases, but only up to certain lethal limits [34]. Previous results indicated that moderate warming (21 °C) enhances the rate of developmental progression and larval dimension in *P. lividus* [20,35], while the highest temperature increase (+6 °C, 24 °C) had a similar effect but also caused a higher proportion of abnormal larvae (30%) [20]. In terms of bioaccumulation, it was previously demonstrated that 1 mM V inhibits skeletogenesis, impeding Ca uptake, while temperature increases the bioaccumulation of both V and Ca [31]. Here, we confirmed the positive correlation between temperature increase and larval size using three metrics of biomineralization: BR, PO, and Pre-O. An additional study showed that a lower V concentration (100 μM) was able to reduce skeleton growth, decreasing another common metric of biomineralization, the body length [21].

In general, as also reported by experimental results obtained in another marine invertebrate (*Mytilus edulis*), the effects on skeletogenesis detected in this paper could be explained by considering that temperature increase changes the solubility and chemical kinetics of the metals involved in the biomineralization process (e.g., calcium) [24].

### 4.3. V and Increased Temperatures Modulated the Metal-Related Proteolytic Activity and the Level of MMPs

Embryo cytomorphology analysis and observations of the total skeletal mass or individual skeletal elements suggest that these parameters could be driven by molecular variations.

In fact, several studies have demonstrated how the combined metals/temperature outcome can affect the antioxidant activity and the detoxification systems [25]. In the experimental model *Mytilus galloprovincialis*, for example, synergic hyperthermia/copper effects on the expression of apoptotic enzymes (caspase-3) or on cytoprotective molecules (HSP 70) have been reported [23].

To our knowledge, apart from these studies, there are not many data on the adaptive capacity of embryonic models triggered by activities that can predispose tissue remodeling.

Based on the results concerning embryo morphology and the quantitative and qualitative analyses of the skeleton, it is possible to hypothesize a correlation with changes in gelatinases, including MMPs. These enzymes, by digesting elements of the extracellular matrix, promote cell progression and tissue reorganization in order to remodel embryo architecture [17,18].

The present study reports that there is a synergistic effect between V and temperature rise in the modulation of these enzymatic activities. Zymography demonstrates that at 24 h, total proteolytic activity is reduced in embryos treated with both V and increased temperature. A reduction in total proteolytic activity is also observed in V-treated embryos at 48 h. On the contrary, gelatinolytic activity increases considerably in untreated embryos as temperature rises.

These data align with previous reports on the kinetics of V-accumulation, which shows a temperature-dependent increase [31]. This accumulation in cells and tissues causes the increase of metal toxic action with repercussions on embryo morphology, skeletal formation, and metal-related proteolytic activity.

Our studies on MMPs show that the course of these gelatinases does not always have the same trend as the total proteolytic activity, but each MMP can behave differently in relation to the stage of development and the toxic insult [17,18].

We have observed that only MMP-9 reflects the total enzymatic activity levels, since in almost all V-exposed embryos its levels are lower than in unexposed embryos at any developmental temperatures. For this reason, we propose the MMP-9-like protein as a marker of response activation in embryos exposed to V and/or to temperature variation during development.

On the other hand, MMP-2 is not drastically modulated either by the effect of V or by the combined effects of V and increased temperature except in embryos developed at 24 °C, both for 24 and 48 h. This result suggests that MMP-2 could be a marker of the effects induced by the dual stress.

MMP-14 in sea urchin embryos has been widely studied, and its role in the remodeling of the extracellular matrix, functional to skeletogenesis, has been reported [17]. This protein has been localized on the filopodia emitted by the primary mesenchyme cells (skeletogenic cells). This enzymatic activity is probably essential for the deposition of the skeletal matrix. The levels of this MMP were high at the gastrula stage (the phase preceding skeletogenesis) and then decreased at the advanced pluteus stage (the phase of completion of the skeleton), confirming its possible involvement in skeletogenesis.

In accordance with the above evidence, the present study reports that in unexposed embryos, MMP-14 is more expressed in gastrulae (embryos grown for 24 h at 18 °C and 21 °C) than in embryos in an advanced developmental stage. In fact, embryos grown at 24 °C, which reach a more advanced developmental stage (pluteus), exhibit a reduction in its amount. This result underlines that the MMP-14 expression level depends on the developmental stage of the embryo.

On the other hand, since all V-treated embryos show a gastrula/prism-like phenotype, they have an MMP-14 level comparable to that of the same developmental stage achieved without V.

As expected, after 48 h of development, the levels of MMP-14 are drastically reduced in almost all the examined embryos. An appreciable expression of MMP-14 can be detected only in V-treated embryos grown at physiological temperature. According to these results, it is possible to assume that V-treated embryos could be in a phase of activation of survival strategies trying to restore the developmental program, maybe modifying the extracellular cell matrix.

Furthermore, these results demonstrate how embryos are highly responsive to the surrounding environment. They are able to activate or inhibit different enzymatic activities, depending on initiated pathways of cell survival or death, in order to safeguard their developmental program [31].

Overall, these results demonstrate how the mechanism of action activated by sea urchin embryos in the presence of a combined metal/temperature stress is very complex. It involves a variation in the kinetics of metal accumulation in a dose/time-dependent manner, and a complex response to the phenomena of cell survival and cell death [31]. However, the adaptive response is complicated as the embryos try to reach an optimal system through the establishment of alternative phenotypes, which allow them to pursue the development program.

These aspects make the molecular processes to be monitored complex, but highlight the mechanisms of action activated by sea urchin embryos in the presence of V and increased environmental temperature.

Our results confirm the choice of a multiple approach to evaluate the impact of stress in order to provide a useful investigation method for environmental management and a formulation of relevant pollution control measures.

## 5. Conclusions

Embryos of marine invertebrates develop in direct contact with the marine environment and their possible pollutants. Therefore, they have settled defense strategies that can induce survival, activating the stress response mechanisms and/or promoting alternative developmental phenotypes [36,37,38,39,40]. The presence of disturbing factors immediately causes a variation in embryonic development that can be detected both at the morphological and molecular levels.

In this study, we expanded our previous results confirming that the increase in temperature worsens the toxic effects induced by V, modulating morphometric endpoints and total gelatinolytic activity and the three gelatinases MMP-2, -9, and -14.

Taken together, our data confirm the *P. lividus* embryo as an excellent bioindicator for the study of chemical pollution and environmental alterations caused by climate change since the interactions among multiple stressors cannot be inferred from the effects of individual stressors alone, highlighting the necessity of adopting comprehensive methodologies to grasp how these stressors interrelate, ultimately aiding in management predictions.

In summary, our findings demonstrate that integrating morphological and morphometric analyses with evaluations of proteolytic activity and MMP-like protein expression yields a substantial response profile to the impacts of both V and temperature.

## Data Availability

The data that support the findings of this study are available on request from the corresponding author.
